# Supplementary material for: Environmental Dispersion of Multiresistant Enterobacteriaceae in Aquatic Ecosystems in an Area of Spain with a High Density of Pig Farming
Source: Antibiotics (Basel). 2025 Jul 25;14(8):753. doi: 10.3390/antibiotics14080753 (PMC12383000; doi:10.3390/antibiotics14080753)
Supplement: Supplementary file 1 [file antibiotics-14-00753-s001.zip › antibiotics-3763397-supplementary.pdf]

Supplementary Section S1.

Location of 11 points where water samples were taken from the rivers Ter, Gurri and Mèder in Osona, Catalonia, Spain

- Vic (Mèder River, five locations): 41º57′20.5″N 2º16′25.8″E; 41º55′46.5″N 2º16′06.2″E; 41º55′28.2″N 2º15′01.0″E; 41º56′21.1″N 2º16′35.2″E; 41º55′45.1″N 2º16′05.6″E
- Manlleu (Ter River, one location): 41º59′55.5″N 2º17′24.3″E
- Roda de Ter (Ter River, one location): 41º59′00.8″N 2º17′59.4″E
- Sant Quirze de Besora (Ter River, one location): 42º06′04.7″N 2º13′05.5″E
- Santa Eugènia de Berga (Gurri River, one location): 41º54′37.1″N2º15′54.0″E
- Torelló (Ter River, one location): 42º06′14.45″N 2º25′48.21″E
- Masies de Voltregà (Ter River, one location): 42º01′11.5″N2º15′05.1″E

Supplementary Section S2.

Table S1. Description of gram-negative bacilli and *Enterobacteriaceae* detected in samples taken from rivers and WWTPs, with genome sequencing

Species	Sampling site	SpaType	ARG(s) present
Aeromonas sobria	WWTP Centelles (influent)	Unknown	blaOXA-392, aadA1, qnrS2, sul1, dfrB5, ere(A), catA1
Aeromonas sobria	WWTP Vic (influent)	Unknown	blaSHV-12, blaFOX-2
Aeromonas sobria	WWTP Viladrau (effluent)	Unknown	blaCEPH-A3
Aeromonas sobria	WWTP Tavernoles (effluent)	Unknown	blaOXA-427
Aeromonas sobria	WWTP Roda de Ter (influent)	1137	blaOXA-427, sul1, aph(6)-Id
Aeromonas sobria	River - Torelló	Unknown	ampS

Aeromonas sobria	River – Sant Quirze Besora	Unknown	blaVCC-1, cphA4
Aeromonas hydrophila	River – Vic	Unknown	blaOXA-427, blaOXA-1, blaCTX-M-15, blaSHV-33, OqxB, aac(6')-Ib-cr, aac(3)-IIa, aph(6)-Id, sul2, fosA6, tet(A), catB3
Aeromonas sobria	River – Sant Vicenç Torelló	Unknown	blaVCC-1, ampS
Aeromonas caviae	River- Manlleu	1137	blaOXA-427, mcr-3.17
Aeromonas sobria	River – Sant Quirze Besora	Unknown	blaFOX-5, ampS, tet(E)
Aeromonas sobria	River – Vic	1952	cphA5
Aeromonas caviae	River – Santa Eugènia de Berga	Unknown	None
Aeromonas salmonicida	River – Roda de Ter	Unknown	None
Aeromonas hydrophila	River – Roda de Ter	Unknown	blaOXA-427
Aeromonas caviae	River – Roda de Ter	321	imiH, ampH
Pseudomonas aeruginosa	River - Vic	Unknown	Unknown
Pseudomonas aeruginosa	River - Vic	252	blaOXA-486, blaPAO, crpP, aph(3')-IIb, fosA, catB7
Pseudomonas aeruginosa	River - Vic	262	blaOXA-488, blaPAO, crpP, aph(3')-IIb, fosA, catB7
Pseudomonas aeruginosa	River - Vic	4249	Unknown
Pseudomonas fluorescens	River- Masies Voltregà	45, 86	None
Pseudomonas fluorescens	River- Vic	16	None
Pseudomonas fluorescens	River- Vic	Unknown	None
Pseudomonas fluorescens	River- Santa Eugènia Berga	63	None
Pseudomonas fluorescens	River – Masies Voltregà	Unknown	None
Pseudomonas fluorescens	River – Manlleu	Unknown	None
Pseudomonas fluorescens	River – Roda de Ter	Unknown	None
Pseudomonas putida	WWTP – Manlleu (influent)	146	None
Pseudomonas putida	WWTP – Tavernoles (influent)	Unknown	None
Pseudomonas putida	River - Vic	Unknown	None

Pseudomonas putida	River- Roda de Ter	168	None
Pseudomonas putida	River- Santa Eugènia de Berga	6	None
Citrobacter freundii	WWTP – Tona (influent)	493	blaCMY-2, blaTEM-1A, qnrB32, aadA2b, sul1
Citrobacter freundii	WWTP – Tona (effluent)	Unknown	blaPAM-1

Table S2. ESBL-producing *E. coli* community human strains

**Nº isolate and residence community of sample provider**	**ST**	**Serotype and FimH**	**ESBL genes**	**Other betalactamase genes**	**Other ARG**	**Plasmid replicons**	**Virulence factors**
P-1 Montesquiu	2179	09:H9; 32	^{bla}CTX-M-65	^{bla}OXA-1, ^{bla}TEM-1B	qnrS2, aac(6')-Ib-cr, sul2, dfrA14, tet(A)	Col (BS512), IncFIB(AP001918), IncFIC(FII)	anr, cea, cma, csgA, cvaC, etsC, fdeC, fimH, fyuA, hlyE, hlyF, iroN, irp2, iss, iucC, iutA, lpfA, nlpI, ompT, sitA, terC, traJ, traT, yehA, yehB, yehC, yehD
P-2 Vic	131	025:H4; 30	^{bla}CTX-M-27		sul1, dfrA17, tet(A)	Col (BS512), Col156, ColRNAI, ColpVC, IncFIA, IncFIB(AP001918), IncFII(pCoo), IncFII(pRSB107), IncN	AslA, anr, cea, chuA, csgA, fdeC, fimH, fyuA, hha, iha, irp2, iss, iucC, iutA, kpsE, kpsMII_K5, nlpI, ompT, papA_F43, sat, senB, sitA, terC, traC, usp, yehA, yehB, yehC, yehD, yfcV
P-3 Tona	131	016:H5; 41	^{bla}CTX-M-15		qnrS1	Col156, IncB/O/K/Z, IncFIB(AP001918), IncFII(29), IncFII(pCoo)	AslA, afaA, afaC, afaD, chuA, cia, csgA, fdeC, fimH, fyuA, hha, iha,irp2, iucC, iutA, kpsE, kpsMII_K5, nfaE, nlpI, ompT, papA_F43, sat, senB, shiA, sitA, terC,

							traJ, traT, usp, yehA, yehB, yehC, yehD
P-4<br><br>Taradell	12150?	016:H5; 41	*bla*CTX-M-15	*bla*TEM-1B		Col156, IncFIB(AP001918), IncFII(29)	AslA, afaA, afaC, AfaD, chuA, csgA, fdeC, fimH, fyuA, hha, iha,irp2, kpsE, kpsMII_K5, nfaE, nlpI, ompT, papA_F43, senB, shiA, sitA, terC, traJ, traT, usp, yehA, yehB, yehC, yehD, yfcV
P-5<br><br>Vic	131	025:H4; 30	*bla*CTX-M-27		sul1, dfrA17, mph(A)	Col(BS512), Col156, IncFIA, IncFIB(AP001918), IncFIB(pLF82-PhagePlasmid), IncFII(pRSB107)	AslA, anr, chuA, csgA, fdeC, fimH, fyuA, gad, hha, iha, irp2, iss, iucC, iutA, kpsE, kpsMII_K5, nlpI, ompT, papA_F43, sat, senB, sitA, terC, traT, usp, yehA, yehB, yehC, yehD, yfcV
P-6<br><br>Calldetenes	744	0101:H9; 54	*bla*CTX-M-14	*bla*TEM-1B	sul1, dfrA17, mph(A), tet(B)	IncI1-I(Alpha)	AslA, chuA, cnf1, csgA, dhaK, fdeC, fimH, fyuA, gad, hha, hlyA, hra, ibeA, iha, irp2, iss, kpsE, kpsMII_K5, mchB, mchC, mchF, nlpI, ompT, papA_F14, papC, shiA, shiB,sitA, terC,usp, yehA, yehB, yehC, yehD, yfcV
P-7<br><br>Tona	131	016:H5; 41	*bla*CTX-M-27	*bla*TEM-1B	aac(3)-IId, sul1, sul2, dfrA17, tet(A)	Col(pHAD28), Col156, IncFIB(AP001918), IncFII(29)	AslA, chuA, cnf1, csgA, fdeC, fimH, fyuA, hha, hlyA, iha, irp2, iucC, iutA, kpsE, kpsMII_K5, nlpI, ompT, papA_F43, papC, sat, senB, shiB, sitA, terC, tia, traJ, traT, usp, yehA, yehB, yehC, yehD, yfcV
P-8<br><br>Tona	131	025:H4; 30	*bla*CTX-M-27			Col156, IncFIA, IncFIB(AP001918), IncFII(pRSB107)	AslA, anr, astA, chuA, csgA, fdeC, fimH, fyuA, gad, hha, hra, iha, irp2, iss, iucC, iutA, kpsE, kpsMII_K5, nlpI, ompT, papA_F43, papC, sat, senB, shiB, sitA, terC, traT, usp, yehA, yehB, yehC, yehD, yfcV

P-9<br><br>L'Esquirol	162	076:H10; 32	blaCTX-M-1	blaTEM-1B	sul2, dfrA17, tet(B)	IncFIB(AP001918), IncFIC(FII), IncFII, IncQ1	anr, csgA,cvaC, etsC, fdeC, fimH, fyuA, hha, hlyE, hlyF, hra, iroN, irp2, iss, iucC, iutA, lpfA, mchF, nlpl, ompT, papC, sitA, terC, traJ, traT, yehA, yehB, yehC, yehD
P-10<br><br>Tona	410	08:H21; 24	blaCTX-M-15		sul2, dfrA17, dfrA36, ant(2")-Ia, mph(A)	IncFIA, IncFIB(AP001918), IncFII	aalH, anr, csgA, etpD, faeC, faeD, faeF, faeH, faeI, fdeC, fimF41, fimH, hha, hlyE, hra, iucC, iutA, lpfA, nlpI, shiA, sitA, terC, tia, traT, yehA, yehB, yehC, yehD
P-11<br><br>Taradell	101	H21; 380	blaCTX-M-32		dfrA1	IncFIB(AP001918), IncFIC(FII), IncI1-I(Alpha)	anr, cea, cma, csgA, etsC, fdeC, fimH, focC, focG, fyuA, gad, hha, hlyE, hlyF, hra, iroN, irp2, iss, iucC, iutA, lpfA, mchB, mchC, mchF, mcmA, nlpI, ompT, papA_F48, papC, sfaD, sitA, terC, traJ, traT, tsh, yehA, yehB, yehC, yehD
P-12<br><br>Vidrà	10	H32; 23	blaSHV-12	blaTEM-1B	dfrA1, tet(A)	IncFII, IncX1	AslA, anr, csgA, fimH, hlyeE, nlpI, terC, traJ, traT, yehA, yehB, yehC, yehD
P-13<br><br>Vic	224	09a:H30; 61	blaCTX-M-15	blaTEM-1B, blaDHA-1	catA1, qnrB4, sul1, erm(B), tet(B)	Col(BS512), Col156, IncFIA, IncFIB(AP001918), IncFIB(pLF82-PhagePlasmid), IncFII(pRSB107), IncFII	anr, astA, csgA, fdeC, fimH, fyuA, gad, hlyE, irp2, iss, nlpI, shiA, terC, tia, traT, yehA, yehB, yehC, yehD
P-14<br><br>Taradell	69	015:H18; 27	blaCTX-M-65	blaOXA-10	qnrS1, dfrA14	IncFIA(HI1), IncFII(pRSB107), IncR	AslA, air, chuA, csgA, eilA, fdeC, fimH, fyuA, gad, hha, hlyE, iha, irp2, iss, kpsE, kpsMII_K5, lpfA, mcbA, nlpI, ompT, papA_F43, sat, sitA, terC, traJ, traT, yehA, yehB, yehC, yehD

P-15 Torelló	12	04:H5; 5	*bla*CTX-M-27		sul1, sul2, dfrA17, mph(A), tet(A)	Col156, IncB/O/K/Z, IncFIA, IncFIB(AP001918), IncFII(pRSB107)	AslA, anr, chuA, cia, clbB, cnf1, csgA, fdeC, fimH, fyuA, gad, hlyA, hra, ireA, irp2, iss, kpsE, kpsMII, nlpI, ompT, papA_F12, papA_F43, papC, senB, shiB, sitA, tcpC, terC, tia, traJ, traT, usp, vat, yehA, yehB, yehC, yehD, yfcV
P-16 Vic	155	09:H10; 24	*bla*CTX-M-65	*bla*TEM-126, *bla*TEM-1B, *bla*TEM-207, *bla*OXA-10, *bla*TEM-30, *bla*TEM-186	aac(3)-IV, qnrS1, sul3, dfrA1, tet(A), cmlA1, catA1, floR	IncFIB(AP001918), IncFII, IncHI2, IncHI2A, IncI1-I(Alpha), IncQ1, IncX4	anr, cma, csgA, cvaC, fdeC, fimH, gad, hha, hlyE, hlyF, iroN, iss, lpfA, nlpI, ompT, sitA, terC, traJ, traT, yehA, yehB, yehC, yehD
P-17 Manlleu (Colombia previously)	131	025:H4; 30	*bla*CTX-M-27		Sul1, sul2, dfrA17, tet(A)	Col156, IncFIA, IncFIB(AP001918), IncFII(pRSB107)	AslA, aamR:FN554766, astA, chuA, cnf1, csgA, fdeC, fimH, fyuA, gad, hha, hlyA, hra, iha, iss, iucC, iutA, kpsE, kpsMII_K5, mchB, mchC, mchF, nlpI, ompT, papA_F19, papA_F20, papC, sat, senB, shiB, sitA, terC, tia, usp, yehA, yehB, yehC, yehD, yfcV
P-18 Masies Roda	3205	09:H30; 54	*bla*CTX-M-32		dfrA5, tet(A), tet(M)	IncFIB(AP001918), IncI1-I(Alpha), IncX1, IncY	anr, cia, csgA, cvaC, etsC, fdeC, fimH, gad, hlyE, hlyF, iroN, iss, iucC, iutA, mchF, nlpl, ompT, sitA, terC, traJ, traT, yehA, yehB, yehC, yehD
P-19 Vic	1193	075:H5; 64	*bla*CTX-M-15		erm(B)	Col(BS512), Col156, IncFIA, IncFIB(AP001918)	AslA, aamR:FN554766, chuA, csgA, fdeC, fimH, fyuA, hra, iha, irp2, iucC, iutA, kpsE, kpsMII_K1, neuC, nlpI, ompT, papA_F43, sat, senB, sitA, terC, usp, vat, yehA, yehB, yehC, yehD, yfcV
P-20	69	086:H18; 27	*bla*CTX-M-15	*bla*TEM-1B, *bla*OXA-1	aac(3)-IIa, aac(6')-Ib-	Col156, IncFIB(K), IncFII(K), IncI2	AslA, air, chuA, clpK1, csgA, eilA, fdeC, fimH,

Vic					cr, sul2, dfrA14, tet(A), catB3		fyuA, gad, hlyE, irp2, kpsE, kpsMII, lpfA, nlpI, ompT, terC, traT, yehB, yehC, yehD
P-21<br><br>Torelló	69	015:H18; 27	blaCTX-M-15	blaTEM-1B	qnrS1, sul2, dfrA7, mph(A)	Col440I, IncB/O/K/Z, IncFIA, IncFIB(AP001918), IncFII, IncQ1	AslA, aamR:FN554766, air, anr, chuA, csgA, eilA, fdeC, fimH, fyuA, gad, hha, hlyA, hlyE, ireA, iroN, irp2, iss, iucC, iutA, kpsE, kpsMII_K52, lpfA, nlpI, ompT, papA_F43, papA_F9, papC, sat, sitA, terC, tia, traJ, traT, yehA, yehB, yehC, yehD
P-22<br><br>Vic	131	025:H4; 30	blaCTX-M-15	blaOXA-1	aac(3)-IIa, aac(6')-Ib-cr, catB3, sul1, dfrA17, mph(A), tet(A)	Col156, Col440II, ColpEC648, IncFIA, IncFIB(AP001918), IncFII(pRSB107), IncP1	AslA, anr, chuA, csgA, fdeC, fimH, fyuA, gad, hha, hra, iha, irp2, iss, iucC, iutA, kpsE, kpsMII_K5, mcbA, nlpI, ompT, papA_F43, papC, sat, senB, shiB, sitA, terC, traT, usp, yehA, yehB, yehC, yehD, yfcV
P-23<br><br>Folgueroles	609	09:H4; 34	blaCTX-M-14	blaTEM-1A	FloR, sul1, dfrA1, mef(C), tet(A)	IncFIB(AP001918), IncFII, IncR	aalH, anr, clpK1, cma, csgA, cvaC, etpD, faeC, faeD, faeF, faeH, faeI, fdeC, fimF41, fimH, gad, hha, hlyE, hlyF, hra, iroN, iss, nlpI, ompT, sitA, terC, traJ, traT, yehA, yehB, yehC, yehD
P-24<br><br>Oristà	131	025:H4; 99	blaCTX-M-27			IncFIA, IncFIB(AP001918), IncFII(pRSB107)	AslA, anr, chuA, csgA, fdeC, fimH, fyuA, gad, hha, hra, iha, irp2, iss, iucC, iutA, kpsE, kpsMII_K5, nlpI, ompT, papA_F19, papA_F20, papC, sat, shiB, sitA, terC, traT, usp, yehA, yehB, yehC, yehD, yfcV
P-25<br><br>Gurb	457	011:H25; 145	blaCTX-M-15		cmlA1, qnrS1, sul3, dfrA12, tet(M)	IncFIA(HI1), IncHI1A, IncHI1B(R27), IncY	AslA, air, chuA, csgA, eilA, espY2:000868321, fdeC, fimH, fyuA, gad, hlyE, irp2, iss, kpsE,

							kpsMII_K5, lpfA, nlpI, ompT, shiB, terC, yehA, yehB, yehC, yehD, yfcV
P-26<br><br>L'Esquirol	131	025:H4; 298		^{bla}TEM-1C	mef(C), tet(A)	IncFIB(AP001918), IncFII, IncI1-I(Alpha)	AslA, anr, cea, chuA, cia, cib, cnf1, csgA, cvaC, dhaK, etsC, fdeC, fimH, fyuA, gad, hlyA, hlyF, hra, ibeA, iroN, irp2, iss, iucC, iutA, kpsE, kpsMII_K5, mchF, nlpI, ompT, papA_F14, papC, shiB, sitA, terC, traJ, traT, usp, yehA, yehB, yehC, yehD, yfcV
P-27<br><br>Torelló	410	H9; 24	^{bla}CMY-2	^{bla}TEM-1A	sul1, dfrA17, mph(A)	IncB/O/K/Z, IncFIB(AP001918), IncFIC(FII)	anr, csgA, cvaC, etsC, fdeC, fimH, fyuA, gad, hha, hlyE, hlyF, iroN, irp2, iss, iucC, iutA, lpfA, mchF, nlpI, ompT, sitA, terC, traJ, traT, yehA, yehB, yehC, yehD
